# Genome-wide identification and expression analysis of the cellulose synthase gene family in potato (*Solanum tuberosum* L.)

**DOI:** 10.3389/fpls.2024.1457958

**Published:** 2024-12-11

**Authors:** Huiling Gong, Junxian Ma, Leonce Dusengemungu, Zaiping Feng

**Affiliations:** ^1^ School of Life Science and Engineering, Lanzhou University of Technology, Lanzhou, China; ^2^ College of Mathematics and Natural Science, The Copperbelt University, Kitwe, Zambia

**Keywords:** potato, *CesA*, biotic stress, expression analysis, qRT-PCR

## Abstract

Potato (*Solanum tuberosum*) is the fourth largest staple food crop globally. However, potato cultivation is frequently challenged by various diseases during planting, significantly impacting both crop quality and yield. Pathogenic microorganisms must first breach the plant’s cell wall to successfully infect potato plants. Cellulose, a polysaccharide carbohydrate, constitutes a significant component of plant cell walls. Within these walls, cellulose synthase (*CesA*) plays a pivotal role in cellulose synthesis. Despite its importance, studies on *StCesAs* (the *CesA* genes in potato) have been limited. In this study, eight *CesA* genes were identified and designated as *StCesA1-8*, building upon the previous nomenclature (*StCesA1-4*). Based on their phylogenetic relationship with Arabidopsis thaliana, these genes were categorized into four clusters (CesA I to CesA IV). The genomic distribution of *StCesAs* spans seven chromosomes. Gene structure analysis revealed that *StCesAs* consist of 12 to 14 exons. Notably, the putative promoter regions harbor numerous biologically functional *cis*-acting regulatory elements, suggesting diverse roles for *StCesAs* in potato growth and development. RNA-seq data further demonstrated distinct expression patterns of *StCesAs* across different tissues. Additionally, quantitative real-time PCR (QRT-PCR) results indicated significant up-regulation of *StCesA5* expression under biotic stresses, implicating its potential involvement in potato disease resistance.

## 1 Introduction

Plant cell wall is a thin, solid and flexible outer cell layer composed of cellulose microfibrils (CMFs) (Cosgrove, 2005), which is known as the ‘first line of defense’ against most pathogenic bacteria in plants. As an important component of plant cell wall, cellulose usually exists in the form of CMFs. The basic structural unit of cellulose is D-glucopyranose, which is linked by *β*-1,4 glycosidic bonds. Cellulose biosynthesis occurs at the plasma membrane. Cellulose synthase complexes (CSCs), a rosette shaped (Doblin et al., 2002), hexamer complex typically composed of 18-36 individual cellulose synthase subunits, synthesize *β*-1, 4-glucan chains using UDP-glucose as substrate to regulate cellulose synthesis (Perrin, 2001; Peng et al., 2002).

*CesA* gene is a gene encoding the catalytic subunit of CSCs, belonging to the GT-A protein family (Liu and Mushegian, 2003). The length of this gene family is generally about 1000bp. The earliest reports of *CesA* genes date back to 1991 and 1995, with the discovery of two *CesA* genes in *Acetobacter xylinus* (Saxena et al., 1991; Saxena and Brown, 1995). Subsequently (Pear et al., 1996), identified two cDNA clones in *Gossypium hirsutum* and one in *Oryza sativa*, marking a significant milestone in plant *CesA* gene research.

Currently, *CesA* gene family members have been identified in various plants, including 10 *CesA* genes in *Arabidopsis thaliana* (Richmond and Somerville, 2000), 12 in *Zea mays* (Appenzeller et al., 2004), 16 in *Solanum lycopersicum* (Song et al., 2019), 26 in *Glycine max* (Nawaz et al., 2017), etc. In *Arabidopsis thaliana*, *AtCesA4* (IRX5), *AtCesA7* (IRX3) and *AtCesA8* (IRX1) are related to the formation of secondary wall (Taylor et al., 2003), while *AtCesA1* (*rsw1*), *AtCesA3* (*ixr1*) and *AtCesA6* (*prc1*) are associated with the primary wall formation (Persson et al., 2007). Additionally, the cellulose synthase interacting protein 1 (*CSI1*) has been shown to interact with *AtCesA1* and *AtCesA6* (Gu et al., 2010), and is highly co-expressed with *AtCesAs* related to the primary wall in Arabidopsis (Zhang and Zhou, 2015). In tomato, the *HEL* gene, homologous to *CSI1*, interacts with *CesA* and influences the spiral growth of the plant (Yang et al., 2020). In rice (Tanaka et al., 2003), identified *OsCesA4*, *OsCesA7* and *OsCesA9* as related to rice secondary wall synthesis through the phylogenetic tree. The insertion of the endogenous retrotransposon *Tos17* revealed that the fragile stem phenotype in rice may be attributed to defects in the secondary wall.

The potato, a staple crop originating from the Andean region, is now widely cultivated with China leading global production (Devaux et al., 2021). Among the various diseases affecting potatoes, late blight, caused by *Phytophthora infestans*, is particularly devastating, often resulting in significant yield losses (Dong and Zhou, 2022). Varieties like ‘*Shepody*’, ‘*Atlantic*’, and ‘*Favorita*’ are highly susceptible, with disease rates soaring above 80% under favorable conditions for the pathogen (Duan et al., 2021). The *CesA* gene plays a crucial role in plant growth and cellulose synthesis, and its variants in potatoes, known as *StCesAs*, are of particular interest. Research indicates that certain *CesA* genes may be involved in susceptibility to late blight (Sun et al., 2016). Therefore, investigating the *StCesA* gene family in potatoes using bioinformatics could unveil their functions and potentially reveal targets for enhancing resistance to late blight. This is vital as breeding for resistance often incorporates resistance genes against *P. infestans (Rpi* genes) from wild relatives, which can be expedited through genetic engineering for durable, broad-spectrum resistance (Paluchowska et al., 2022). Understanding the *StCesA* genes’ involvement in late blight could lead to more resilient potato cultivars, addressing a major agricultural challenge.

## 2 Materials and methods

### 2.1 Plant materials and treatments

The potato hybrid resistant cultivar ‘*Longshu* 10’ was subjected to a biotic stress experiment using the oomycete pathogen Phytophthora infestans, the causative agent of late blight. The plant were cultured in the Plant Tissue Culture Laboratory of Lanzhou University of Technology. Tubers of uniform size were selected for growth buried in pots 20 cm in diameter, with a nutrient soil to vermiculite ratio of 3:2, a light/dark cycle of 16/8 h, a day/night temperature of 23 ± 2°C, and weekly watered. After approximately 4 months, potato plants with similar growth were selected, and the fourth and fifth compound leaves below the apical leaves were removed for leaf inoculation *in vitro*. The leaves were uniformly sprayed with spore suspension of *P. infestans* at a concentration of 1×10^5^ cells/mL, while the control group received sterile water instead. The inoculated leaves were located at 18 to 20°C and 100% humidity. Samples were collected at 0, 24, 48, and 72 hours post-inoculation to assess the expression patterns of *StCesA* genes under this specific biotic stress condition. Three biological replicates were performed at each time point to ensure statistical validity. The samples were promptly frozen in liquid nitrogen and stored at −80°C until further analysis.

### 2.2 RNA isolation, cDNA synthesis, and qRT-PCR

Total RNA of leaves was extracted using TRIzol Reagent (Sangon Biotech, China), and the operation procedure was performed according to the manufacturer’s protocol. After the extraction, first-strand cDNA was synthesized from 2μg of total RNA using PrimeScript™ RT Master Mix (Perfect Real Time)(Takara, Japan). Specific primers were designed using Primer Premier 5 (Singh et al., 1998) and synthesized commercially (Sangon Biotech, Shanghai, China). *StEF-1α* (LOC102600998) was used as the reference gene for qRT-PCR in a 10μL master mix: 5 µL of 2×TB Green Premix Ex Taq II (Tli Rnase H Plus) (Takara, Japan), 1 µL of the template, 3.6 µL of ddH_2_O, and 0.2 µl (10μM) of each primer. The qRT-PCR program was: (і) predenaturation at 95°C for 2min, (ii) 40 cycles of amplification: denaturation at 95°C for 5s, annealing at 55°C for 30s, extension at 72°C for 1min, and (iii) extension at 72°C for 10min. The expression levels of genes were analyzed using the 2^−ΔΔCT^ method (Livak and Schmittgen, 2001). SPSS was used to analyze the results for significant differences, and the relative gene expression levels were visualized by GraphPad Prism 9.

### 2.3 Identification of *CesA* gene family in potato

Sequence files and other related information (Hardigan et al., 2016) of the doubled monoploid (DM) *S. tuberosum* were obtained from PGSC (http://spuddb.uga.edu/). Sequence of *AtCesAs* were from TAIR (https://www.arabidopsis.org/). The Hidden Markov Models (HMMs) of *Cellulose_synt* and *zf - UDP* (PF03552 and PF14569) were downloaded from Pfam (http://www.ebi.ac.uk/interpro/entry/pfam/) to identify *StCesAs*. Firstly, PF03552 and PF14569 were used as templates to search the potato protein sequence (E-value ≤ 1e^-5^) by HMMER v3.4 (http://hmmer.org/), and the candidate members of *StCesA* gene family were obtained, which were recorded as G1. Then, 10 known *AtCesA* protein sequences were used for BLAST alignment in the potato genome database (E-value ≤ 1e^-5^), and the obtained genes were recorded as G2. The genes of G1 and G2 were merged and compared, then duplicate genes were deleted. Finally using NCBI-CDD (https://www.ncbi.nlm.nih.gov/Structure/bwrpsb/bwrpsb.cgi) and SMART (http://smart.embl-heidelberg.de/) to eliminate the sequences without typical domain of CesA proteins. Eight *StCesA* gene family members were finally determined and named as *StCesA1*-*StCesA8* on the basis of retaining the original gene names (Oomen et al., 2004).

### 2.4 Phylogenetic analysis of the *CesA* family genes in potato

The 10 known *AtCesA* protein sequences and the above 8 *StCesA* protein sequences were aligned by the ClustalW algorithm in MEGA v11.0.13 (Tamura et al., 2021) (https://www.megasoftware.net/) and the NJ (Neighbor-Joining) tree was constructed. The test of phylogeny is bootstrap method and 1000 bootstrap replications were chosen. The phylogenetic tree was generated using iTOL v6.9 (Letunic and Bork, 2024) (https://itol.embl.de/) online server.

### 2.5 Gene structure, *motif* distribution, and sequence analysis

In order to analyze the gene structures, the online website GSDS 2.0 (Hu et al., 2015) (http://gsds.gao-lab.org/) was used to analyze the gene structures of *StCesAs* according to the CDS sequence and genome sequence, and the gene structure map was drawn. To analyze *motifs*, use MEME v5.5.4 (Bailey et al., 2015) (https://meme-suite.org/meme/tools/meme) to predict the *motifs* of *StCesA* proteins, the number of *motifs* is set to 10, the rest of the parameters of the same by default. For understanding physical and chemical properties of *StCesA* proteins, using Expasy ProtParam tool (https://web.expasy.org/protparam/) to predict relative molecular weight, isoelectric point, hydrophilicity, and other characteristics.

### 2.6 Chromosomal distribution, prediction of subcellular localization, and synteny visualization

In order to understand the distributions of *StCesAs* on chromosomes, the MG2C-2.1 tool (Chao et al., 2021) (http://mg2c.iask.in/mg2c_v2.1/) was used to map the location of *StCesA* genes on chromosomes according to the annotation file. For predicting the probable subcellular locations of *StCesA* proteins, we used WoLF PSORT program (Horton et al., 2007) (https://www.genscript.com/wolf-psort.html). Synteny analysis of the *CesA* genes in Arabidopsis, potato and tomato was performed to determine the synteny relationship between them. The figure was generated using the MCScanX algorithm and the Dual Synteny Plot program of TBtools (Chen et al., 2023).

### 2.7 Gene ontology enrichment and analysis of *cis*-acting regulatory elements in promoter regions

GO (Gene Ontology) enrichment of *StCesA* protein sequence was performed by STRING (https://cn.string-db.org/).The 2000 bp sequences upstream of the transcription start site of each *StCesA* gene were selected as promoter sequences and submitted to PlantCARE (Lescot et al., 2002) (https://bioinformatics.psb.ugent.be/webtools/plantcare/html/) to predict *cis*-acting regulatory elements with biological functions. The analysis results were visualized using the ‘ggplot2’ package in R.

### 2.8 *In-slico* expression of *StCesAs* in different tissues

The RNA-seq data of different tissues was derived from PGSC database (NCBI accession: SRA030516) (Potato Genome Sequencing et al., 2011). The gene IDs of *StCesAs* were retrieved from the database, and the FPKM(Fragments Per Kilobase of exon model per Million mapped fragments) (Mortazavi et al., 2008) values of leaves, roots, flowers, petals, buds, sepals, stolons and petioles were retrieved. The FPKM values were log10 transformed, and the heat map of relative gene expression level was generated by R.

## 3 Results

### 3.1 Identification of *CesA* gene family in potato

A total of 8 *CesA* genes were identified in potato. Information on *StCesA* proteins and their physical and chemical properties (**Table 1**) indicates that *StCesA* family members encode proteins with amino acid lengths ranging from 998 to 1091 aa. The minimum molecular weight (MW) is 113.37kDa and the maximum is 123.28kDa. The theoretical isoelectric point (pI) ranges from 6.35 to 7.07. The grand average of hydropathicity score (GRAVY) is between -0.195 and -0.110, indicating that the proteins are hydrophilic. Subcellular localization prediction results showed that *StCesA* proteins were all expressed on the plasma membrane.

**Table 1:** The gene and protein characteristics of the *StCesA* gene family in *S. tuberosum*.

Gene name	Transcript ID	AA	MW(kDa)	pI	GRAVY	Chr.	Start	End
*StCesA1*	PGSC0003DMT400017056	1091	122819.49	7.05	-0.195	4	60250478	60257545
*StCesA2*	PGSC0003DMT400053572	1086	121708.43	6.39	-0.195	8	32234666	32242964
*StCesA3*	PGSC0003DMT400017535	1083	120886.57	6.99	-0.171	1	65771775	65780089
*StCesA4*	PGSC0003DMT400027996	1084	121822.55	7.07	-0.169	12	54535536	54543099
*StCesA5*	PGSC0003DMT400010147	1090	122531.85	6.36	-0.144	11	7327209	7334866
*StCesA6*	PGSC0003DMT400024518	1084	123276.79	6.5	-0.177	4	62605488	62610064
*StCesA7*	PGSC0003DMT400028958	1041	117491.16	6.44	-0.189	7	1104236	1110318
*StCesA8*	PGSC0003DMT400073084	998	113370.33	6.35	-0.110	2	31665393	31670190

### 3.2 Phylogenetic analysis

**Figure 1:**
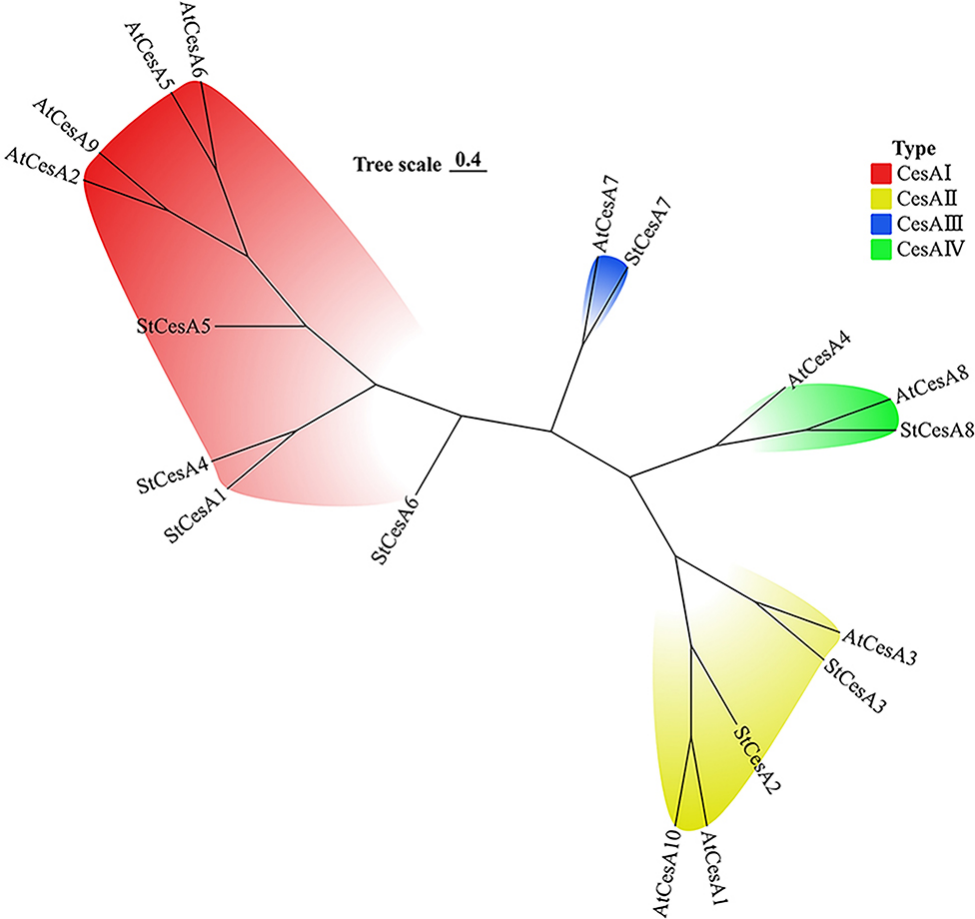
Phylogenetic analysis of *CesA* proteins from *A. thaliana* and *S. tuberosum*.

The phylogenetic tree was constructed by multiple comparison of 10 *AtCesAs* and 8 *StCesAs*. *CesA* gene family members are divided into 4 clusters (**Figure 1**): *CesA* I cluster contains 4 *StCesAs* and 4 *AtCesAs*, CesA II cluster contains 2 *StCesAs* and 3 *AtCesAs*, and *CesA* III cluster contains 1 *StCesA* and 1 *AtCesA*. The CesA IV cluster contains 1 *StCesA* and 2 *AtCesAs*.

### 3.3 Gene structure and *motif* distribution of *StCesAs*

**Figure 2:**
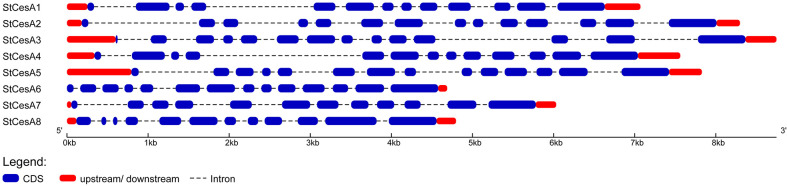
Exon-intron structures of 8 *StCesAs* genes. The blue boxes indicate exons, dashed lines indicate introns, and upstream or downstream regions are indicated by red boxes.

By analyzing the structures of *StCesAs* through GSDS online server, the distributions of exons and introns of *StCesAs* was determined. There is no significant difference in the number of exons and introns of *StCesAs*. *StCesA1* and *StCesA4* contain 13 exons and 12 introns, followed by *StCesA2*, *StCesA3*, *StCesA5* and *StCesA6* contain 14 exons and 13 introns. *StCesA7* and *StCesA8* contain 12 exons and 11 introns (**Figure 2**).

**Figure 3:**
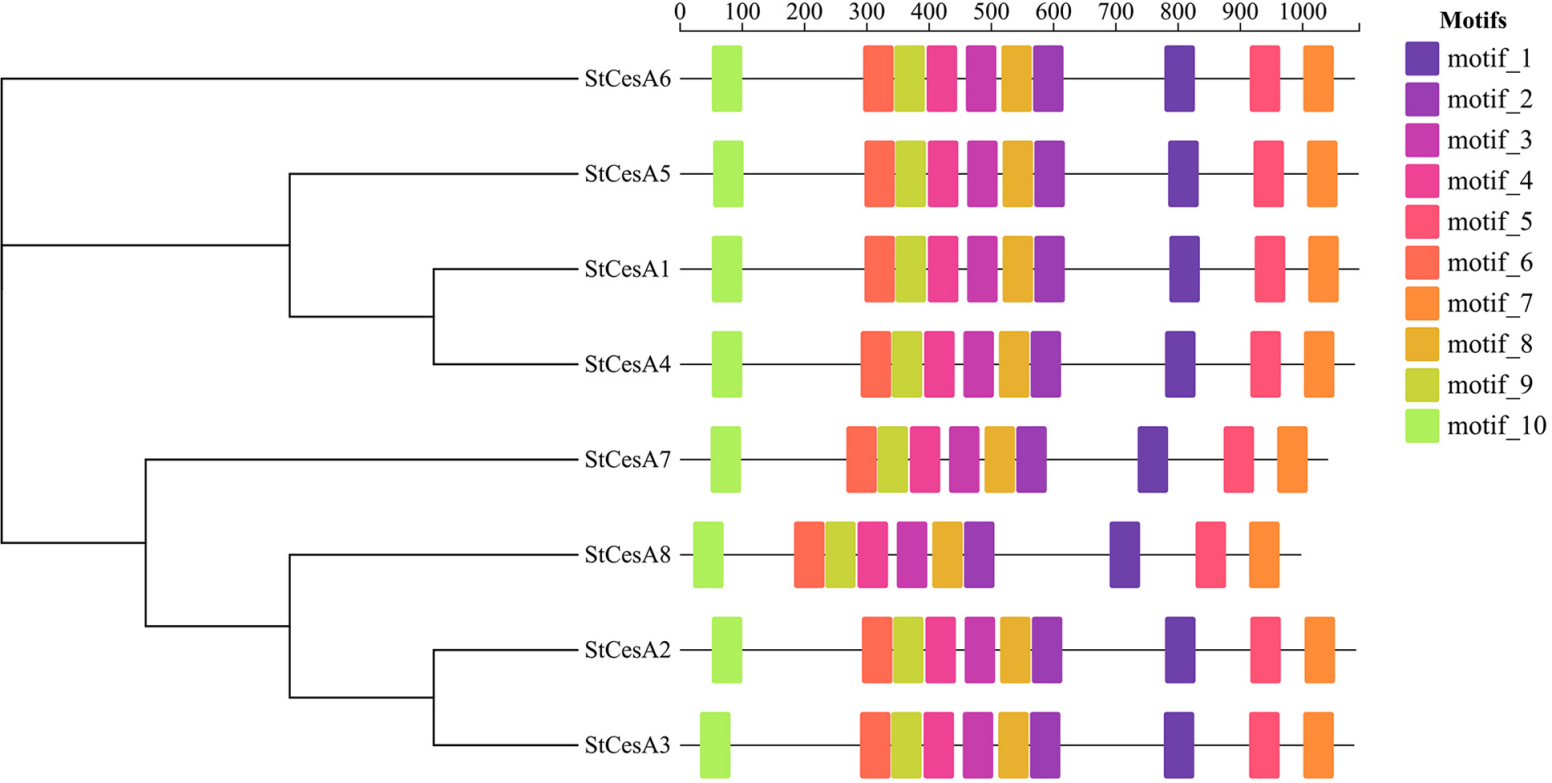
*Motif* analysis of *StCesAs* was predicted using the MEME online server.

In order to further explore the structures and functions of *StCesA* proteins, 10 *motifs* were predicted using MEME Suite online server, and they were found to have a relatively consistent arrangement and distribution (**Figure 3**), indicating a relatively close relationship, which is helpful to study the evolutionary link between CesA proteins.

### 3.4 Chromosomal distribution and synteny analysis

**Figure 4:**
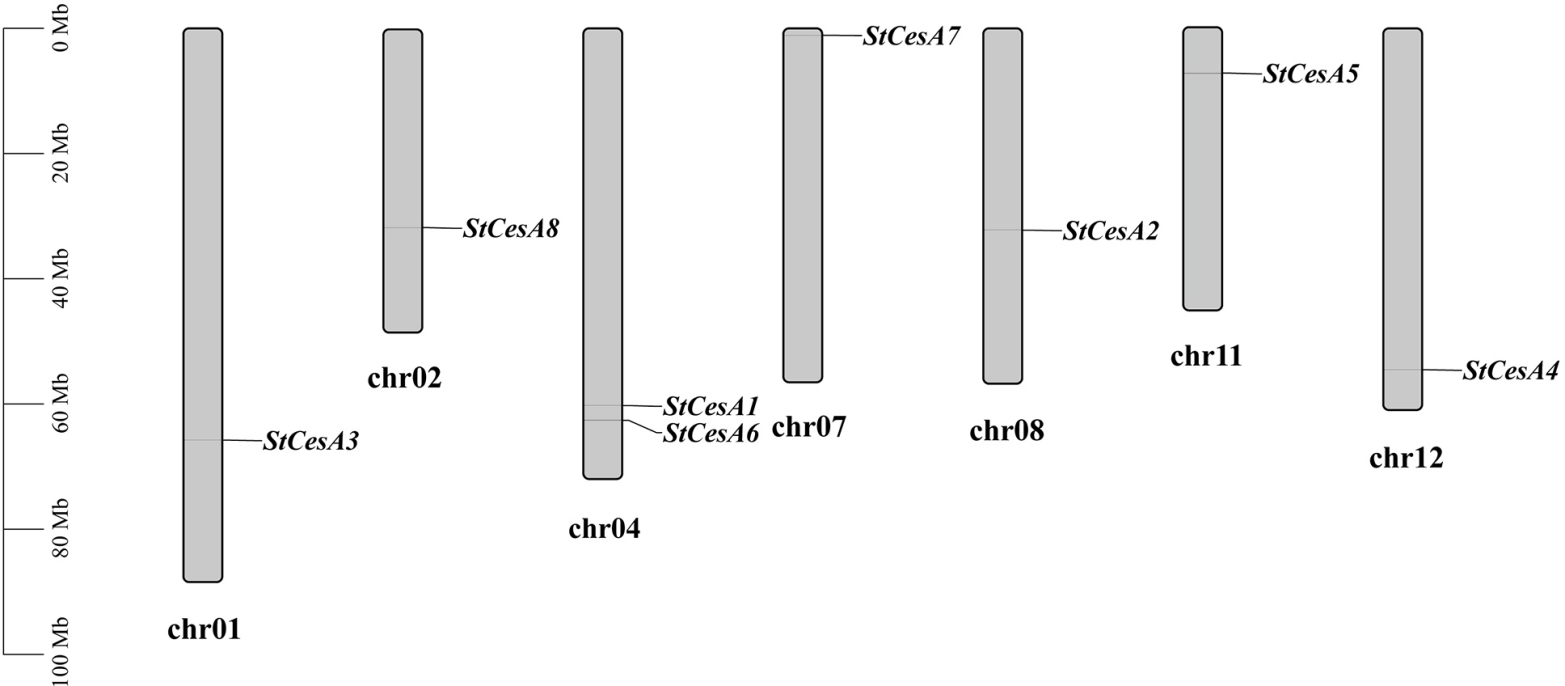
Localization of *StCesA* genes on chromosome.

**Figure 5:**
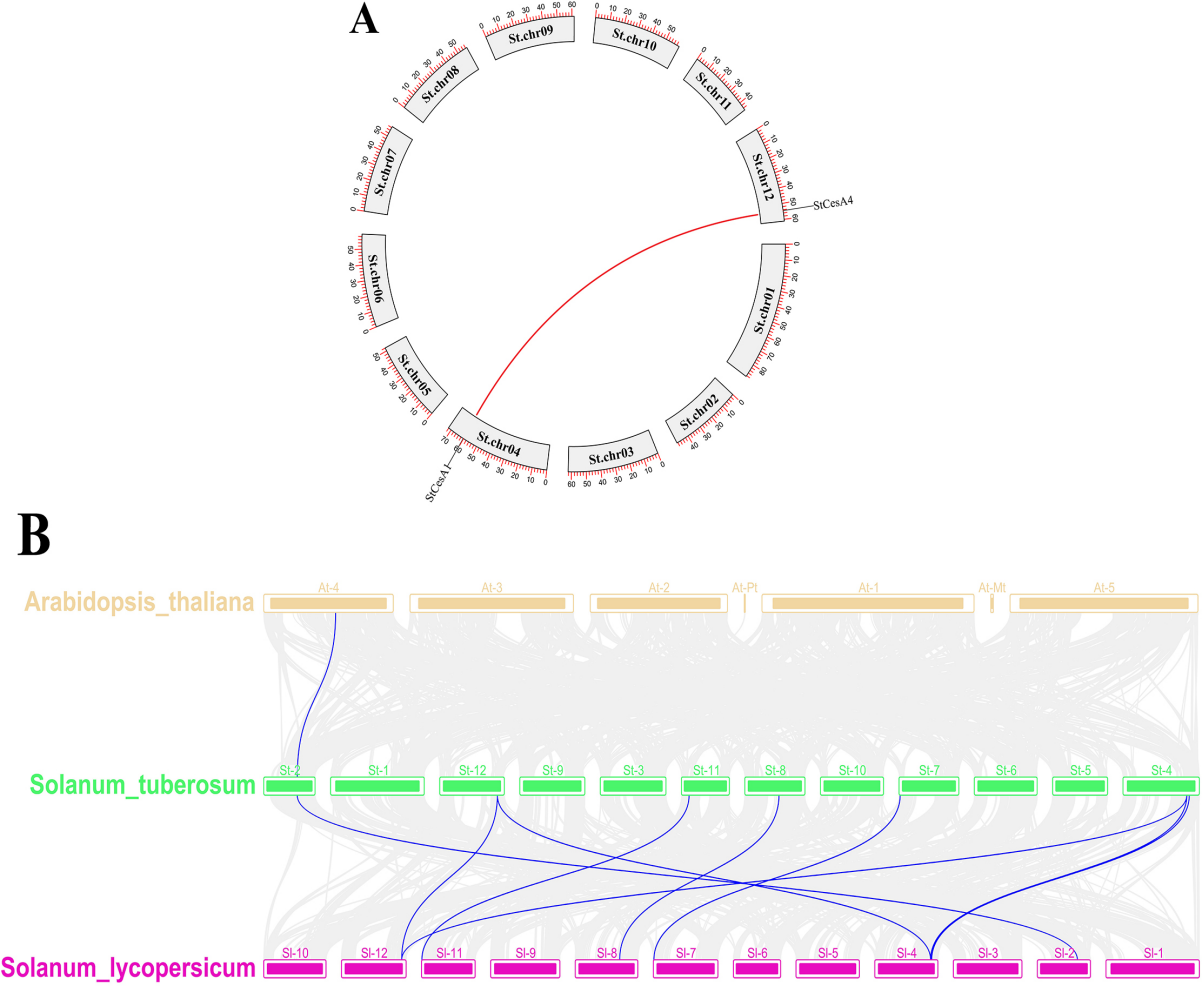
**(A)** Duplicate events of the *CesA* genes in the potato genome. **(B)** Synteny analysis of *CesA* genes between *A thaliana*, *S. tuberosum* and *S. lycopersicum*. Collinear blocks are shown with grey and the collinear *CesA* genes between species are represented by blue lines.

Eight *StCesAs* were scattered on chr1, chr2, chr4, chr7, chr8, chr11 and chr12 (**Figure 4**). There are two on chr4 but none on chr3, chr5, chr6, chr9, and chr10. In plant genome evolution, tandem and segmental duplications contribute to the expansion of gene families with new members and new functions (Bano et al., 2021b). Intraspecies synteny analysis showed that *StCesA1* and *StCesA4* have a segmental duplication (**Figure 5A**). Interspecies synteny analysis was performed between Arabidopsis and potato and there is a gene duplication (*AT4G18780* and *StCesA8*). Synteny analysis was also performed between the potato genome and the genome of tomato, a plant from the same family (**Figure 5B**), because late blight occurs mostly in them. A total of 10 homologous gene pairs were found between *StCesAs* and *SlCesAs* (both *StCesA1* and *StCesA4* were homologous to *Solyc12g056580.2.1* and *Solyc04g071650.3.1*). The structures and functions of *StCesAs* and *SlCesAs* may be highly similar, which provides a new direction for further studying the functions of cellulose synthase genes in potato and tomato.

### 3.5 Gene ontology enrichment of *StCesA* genes

**Figure 6:**
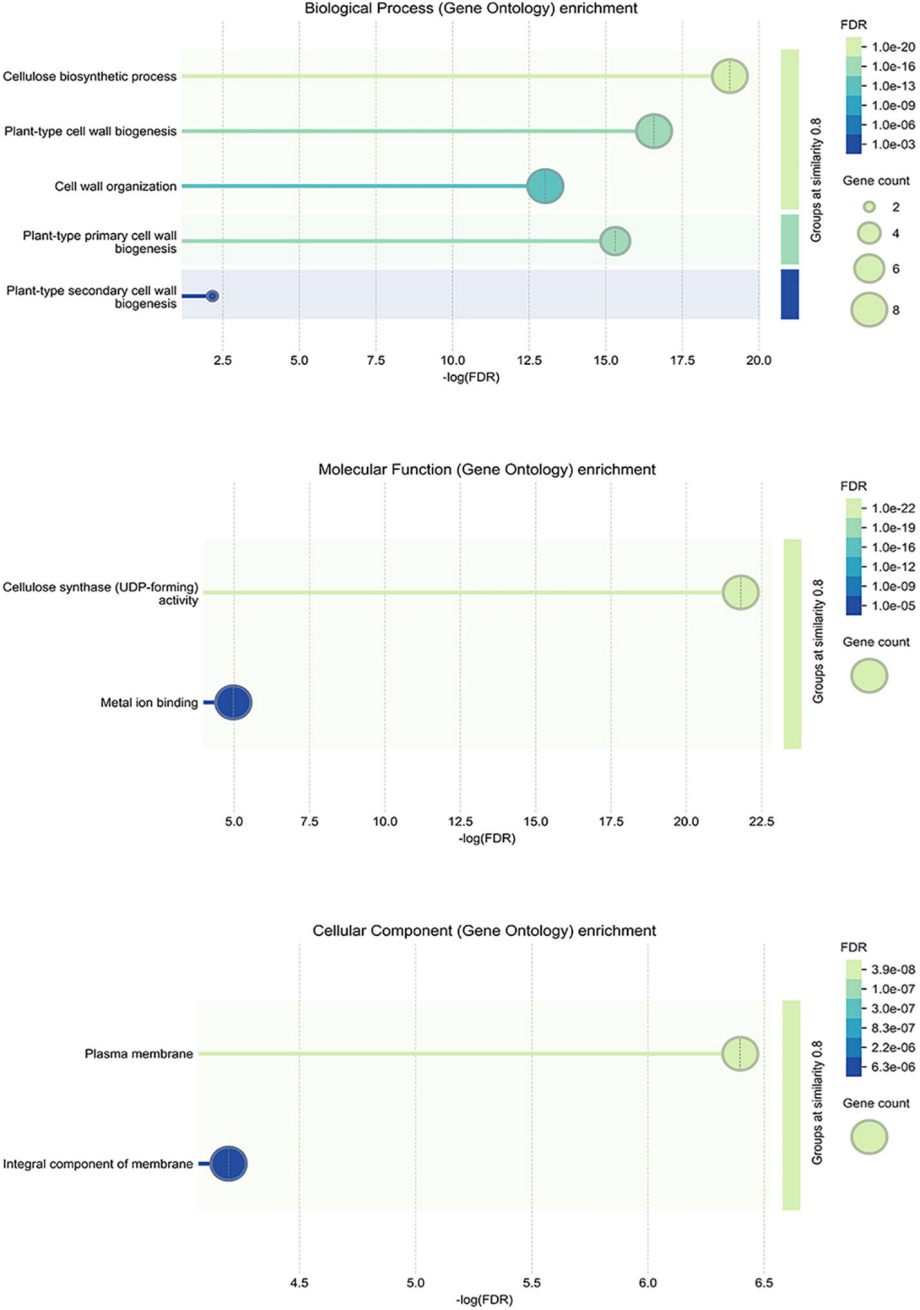
GO enrichment analysis of *StCesA* genes in *S. tuberosum*. It contains three parts: biological process, molecular function and cellular component.

GO enrichment pathway analysis was performed from three aspects (**Figure 6**). The results suggest that *StCesA* genes play a number of important functions in potato. In the Biological Process, most *StCesA* genes play a role in cellulose biosynthetic, plant-type cell wall biogenesis, etc. In the Molecular Function, in addition to their cellulose synthase (UDP-forming) activity, they also involve in metal ion binding, which is worth noting. In the Cellular Component, all members are inseparable from the membrane.

### 3.6 Analysis of *cis*-acting regulatory elements in promoter regions

**Figure 7:**
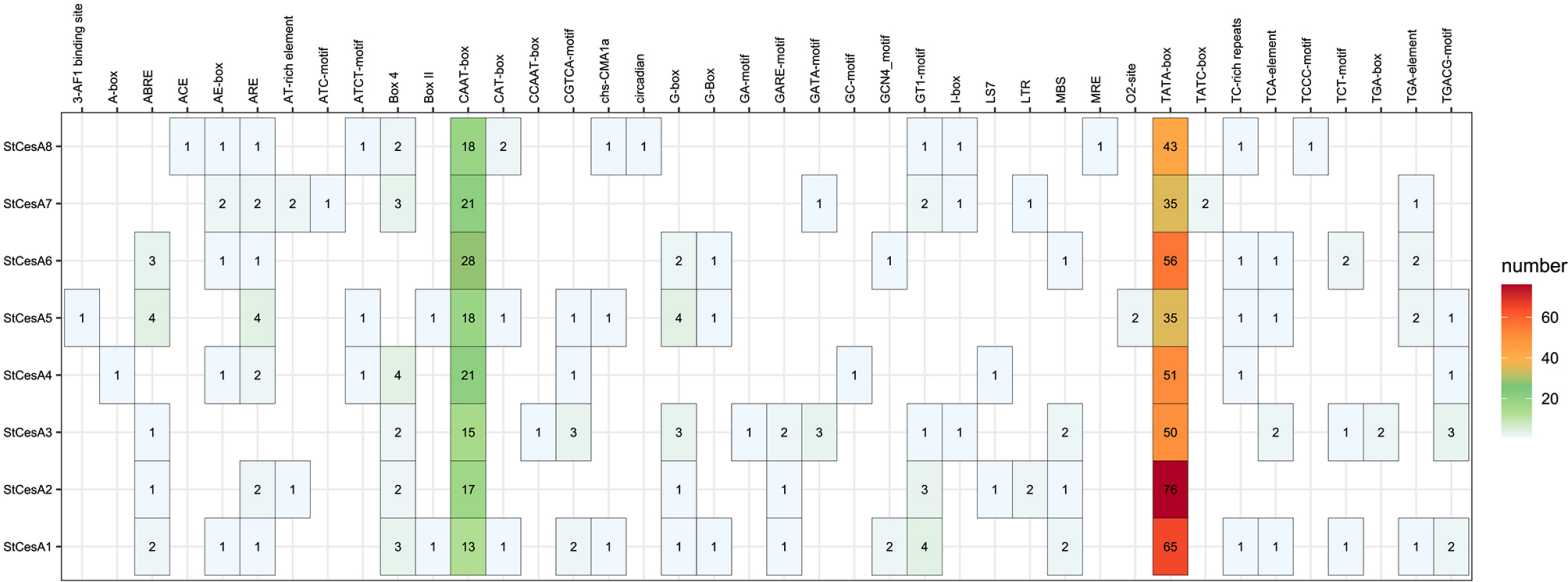
*Cis*-acting regulatory elements in 2000bp promoter region upstream of *StCesAs*.

We identified 40 different *cis*-acting regulatory elements in the promoter regions of the *StCesAs* (**Figure 7**). In addition to the common CAAT-box and TATA-box, the *cis*-acting regulatory elements of *StCesAs* also include abscisic acid response elements (ABRE), methyl jasmonate response elements (CGTCA-*motif*, TGACG-*motif*), light signal response elements (G-box, Box 4, GT1-*motif*), antioxidant response elements (ARE), etc. In addition, there are TC-rich repeats, which are elements involved in disease resistance and stress response. To sum up, *StCesAs* play an important role in potato growth under both abiotic and biotic stress.

### 3.7 *In-slico* expression of *StCesAs* in different tissues

**Figure 8:**
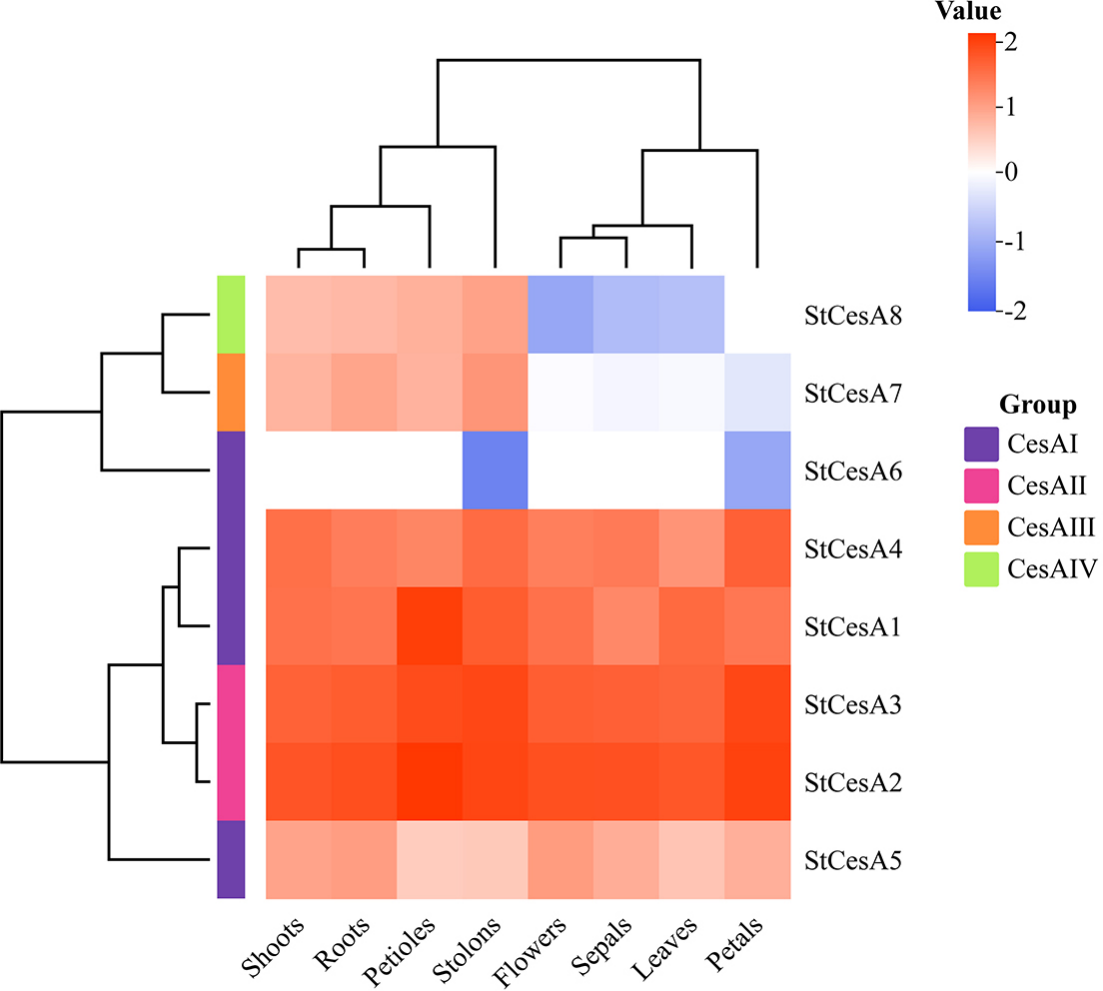
*In-silico* expression analysis of *StCesAs* in 8 tissues of *S. tuberosum.* The relative expression levels of 8 *StCesAs* are standardized by transforming to Log_2_ format.

In order to understand the expression pattern of *StCesAs* in different tissues, RNA-Seq data of leaf, root, flower, petal, bud, sepal, stolon, and petiole was downloaded from the potato transcriptome database. The results were visualized using the ‘ggplot2’ package in R (**Figure 8**). The expression of *StCesA2* was the highest in all eight tissues, while *StCesA6* was only weakly expressed in stolons and petals, and *StCesA8* was not expressed in petals. By analyzing the expression levels of each member, the expression levels of *StCesA1* and *StCesA2* were the highest in petioles, *StCesA3*, *StCesA7* and *StCesA8* were the highest in stolons, *StCesA4* and *StCesA6* were the highest in petals, and *StCesA5* was the highest in flowers.

### 3.8 Expression patterns of *StCesA* genes in response to biotic stress

**Figure 9:**
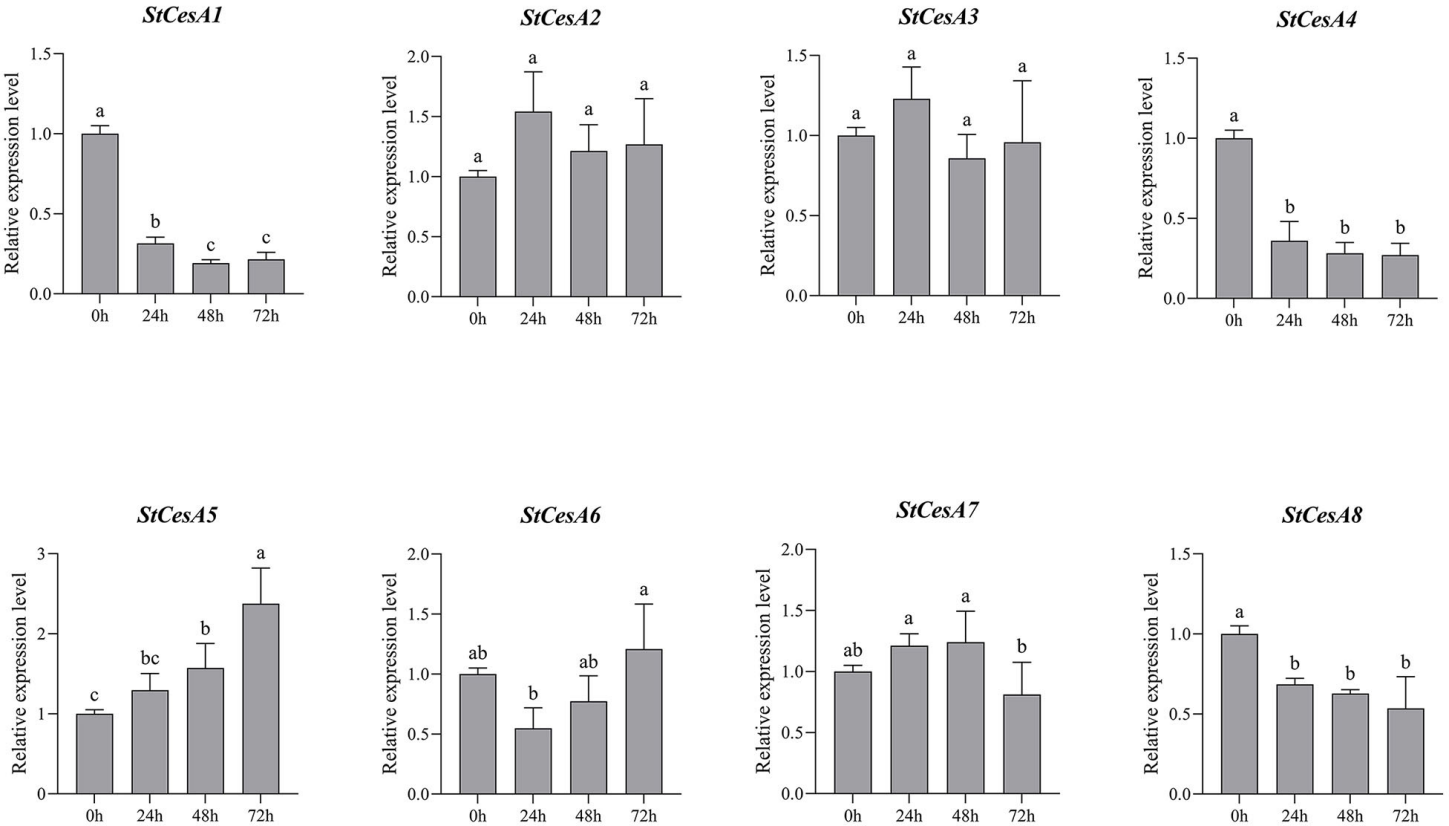
Relative expression levels of 8 *StCesA* genes in *S. tuberosum* under *P. infestans* stress. Data are expressed as mean ±SEM. Different lowercase letters indicate significant differences at different time points (p < 0.05).

To understand the changes in the relative expression of *StCesAs* (0h, 24h, 48h and 72h) in potato leaves infected by *P. infestans*, we performed qRT-PCR analysis of 8 *StCesA* genes (**Figure 9**). For ‘*Longshu* 10’, 72h after infection, the relative expression level of *StCesA1*, *StCesA4*, *StCesA5* and *StCesA8* changed significantly, while *StCesA2*, *StCesA3*, *StCesA4* and *StCesA6* showed no significant change. 0~24h, the relative expression level of *StCesA1、4、8* was significantly downregulated; 24~48h, only *StCesA1* was significantly downregulated;48~72h, *StCesA5* was highly upregulated, but the relative expression level of *StCesA7* was significantly downregulated. Overall, the relative expression level of *StCesA5* increased with time after stress. At the same time, there was no significant difference between the relative expression level of *StCesA2* and *StCesA3*. The pattern of changes in the relative expression level of *StCesA1* and *StCesA4* after stress was the same, which is another strong evidence of their high homology.

## 4 Discussions

Cellulose is one of the important components of the plant cell wall, and its biosynthesis is mainly related to cellulose synthase (*CesA*), KORRIGAN (KOR) (Zuo et al., 2000), sucrose synthase (*SuSy*) (Cardini et al., 1955) and other enzymes involved in the process. Since the first *CesA* gene was reported, *CesA* genes have been identified in many plants such as *Arabidopsis thaliana*, tobacco, tomato, barley, and maize (Richmond and Somerville, 2000; Appenzeller et al., 2004; Burton et al., 2008; Song et al., 2019). These studies contribute to our understanding of the structure, function, and evolution of the *CesA* gene family in plants. The continuous updates to the potato genome database have facilitated the study of gene families with specific functions. In this study, we identified eight *CesA* family members in potato, laying the foundation for future functional analyses of these genes.

The amino acid sequence length of each *StCesA* protein was similar, about 1000 aa (**Table 1**). There were no significant differences in physicochemical properties such as MW, pI, and GRAVY between them, indicating no high variation among *StCesAs*. In order to understand the possible interaction between *StCesA* protein and plant system, the subcellular localizations of *StCesA* proteins were predicted. The results showed that all *StCesA* proteins were localized on the plasma membrane, confirming their “frontier” positions in biotic and abiotic stresses. Our findings correlates with the results from similar studies in other plant species. For instance, research on maize has identified distinct *CesA* cDNAs and has shown that *CesA* genes are expressed in various organs, with some genes being specific to certain cell types involved in primary or secondary wall synthesis (Holland et al., 2000). This suggests that while there may be a conserved core function among *CesA* genes, their expression patterns and roles can vary significantly between species. As *AtCesA1*, *AtCesA3*, *AtCesA6* were associated with the formation of the primary wall, and *AtCesA4*, *AtCesA7*, *AtCesA8* were related to the formation of the secondary wall. We speculate that *StCesA2* and *StCesA3* are related to the formation of the potato primary wall. In contrast, *StCesA7* and *StCesA8* are associated with the formation of the potato secondary wall, and the results need to be verified by experiments.Moreover, studies on sweet potato have revealed that sucrose synthase genes, which are part of a different but related gene family, undergo segmental and tandem duplications during evolution and are highly expressed in sink organs (Zhou et al., 2024).

*StCesA* genes have 12~14 exons and encode proteins of 998~1091 aa in length (**Figure 2**), while *AtCesA* genes have 10~14 exons and encode proteins of 985~1088 aa. Introns play important functions in the evolution of some plant species (Roy and Gilbert, 2006). In the evolutionary analysis of *CesA* genes, the exon-intron structure distribution of *StCesA* genes and *AtCesA* genes is similar, most of the genes were highly conserved during evolution, and the introns of these genes were not lost during evolution, while the introns of some other genes would be lost over evolutionary time (Bano et al., 2021a). In general, the genomes of more advanced species comprise fewer introns (Roy and Gilbert, 2005). These results indicate that the *CesAs* gene structure is evolutionarily conserved in higher plants.

**Figure 10:**
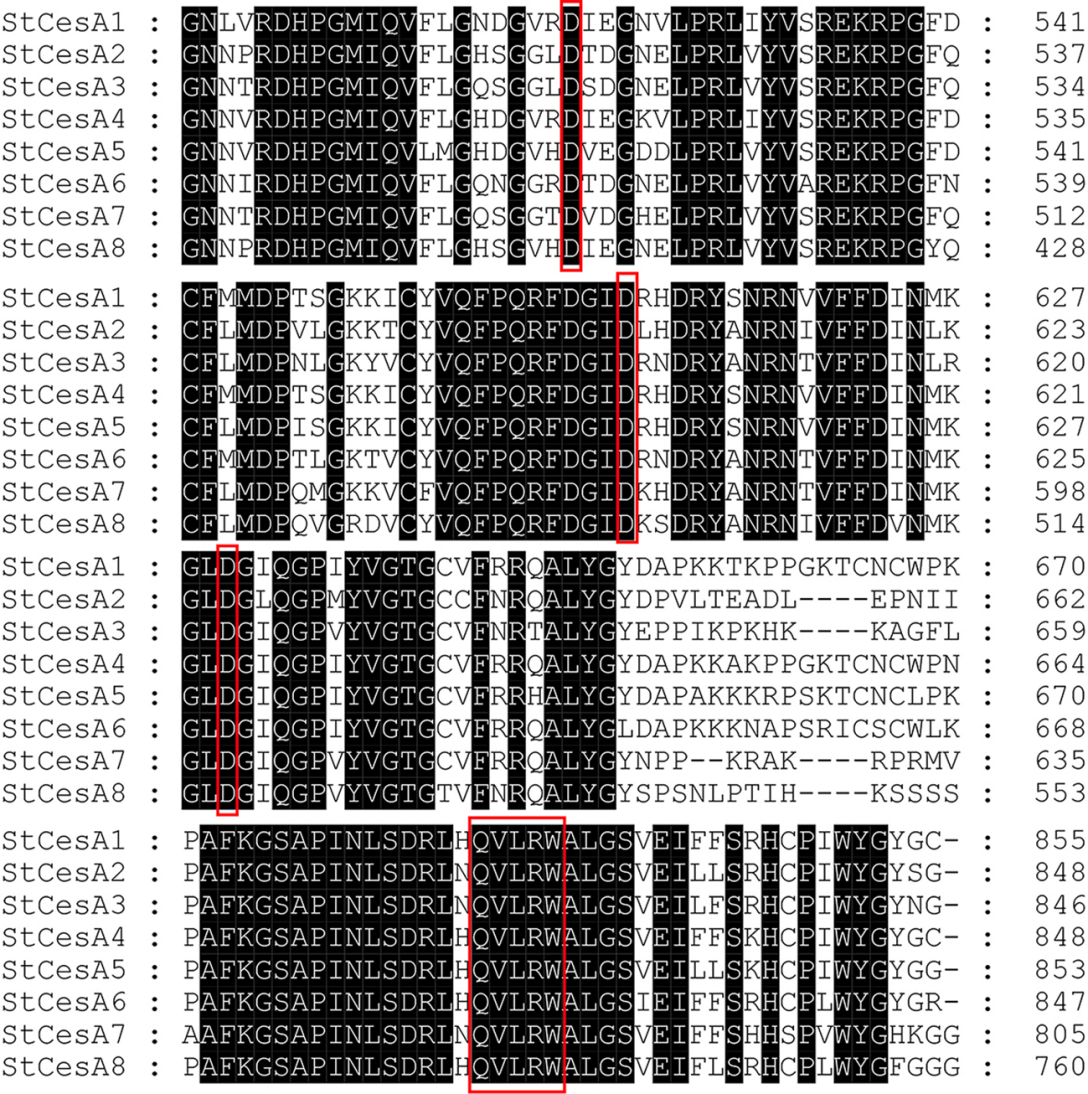
Amino acid sequence alignment of *StCesA* gene family proteins. The black parts represent the identical amino acid residues. The red boxes represent the typical “D, D, D, QXXRW” conserved domain of the *CesA* gene family.

*CesA* proteins in plants have eight transmembrane domains, two located near the N-terminal, and the other six clustered near the C-terminal. The middle region is a hydrophilic intracellular region, and there is a conserved structure of “D, D, D, QXXRW” in this region (**Figure 10**), which is closely related to the catalytic activity, as is *StCesA* proteins. Each member of the *CesA* family has a cysteine-rich, conserved CXXC domain at the N-terminus, which can fold into a zinc finger or LIM transcription factor conformation (Baltz et al., 1992)—starting from amino acids 10 to 40 at the N-terminal. Its base sequence for CX_2_CX_12_FXACX_2_CX_2_PXCX_2_CXEX_5_GX_3_CX_2_C (Somerville, 2006). When the oxidation condition of zinc finger domain is changed, the stability of CSC may be destroyed and the synthesis of crystalline cellulose may be inhibited (Kurek et al., 2002).

Potato and tomato are of the same family and have high homology. According to the localization of genes on chromosomes and synteny analysis, chromosome rearrangements exist in the genome differentiation of potato and tomato. The physical map showed that the *StCesA* gene also rearranged with chromosomes, and the *CesA* gene family had exemplary conservation in potato and tomato genomes.

Analysis of *cis*-regulatory regulatory elements of the promoter sequence (2000bp) of members of the *StCesA* family (**Figure 7**) revealed that all members contain multiple *cis*-acting regulatory elements in response to adversity stress. Biotic and abiotic stress can stimulate the synthesis of methyl jasmonate and ethylene, thereby inducing the expression of genes involved in stress response and enhancing defense response. *AtCesA3* deletion mutants have constitutive expression of stress response genes, increase methyl jasmonate and ethylene synthesis, and to improve resistance to fungal pathogens (Ellis et al., 2002). *StCesA1*, *StCesA3*, *StCesA4*, and *StCesA5* have elements in response to methyl jasmonate, and it is speculated that they may participate in the reaction regulated by methyl jasmonate. It has been shown that altering the integrity of the secondary wall by inhibiting cellulose synthesis leads to the specific activation of new defense pathways, thereby creating an antimicrobial-enriched environment (e.g., peptides, PR proteins, and secondary metabolites) that is harmful to pathogens (Hernandez-Blanco et al., 2007). In addition, the promoters of all *StCesA* genes contain elements related to light response, suggesting that light may induce *StCesA* gene expression.

**Figure 11:**
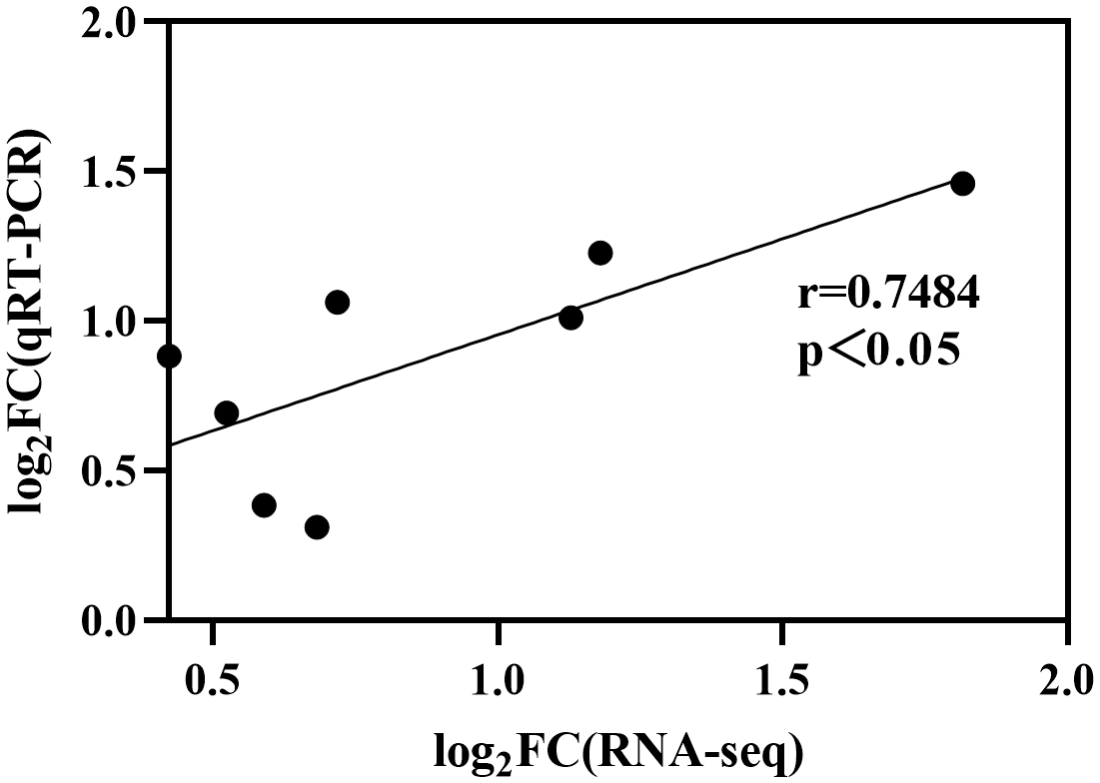
Differences in gene expression between RNA-seq and qRT-PCR. Data was transformed to Log_2_ format and significantly correlated (r=0.7484, p<0.05).

The expression of *StCesAs* was analyzed using RNA-seq data (**Figure 8**). Under normal conditions, some genes (*StCesA1*, *2*, *3*, *4*, *5*) were highly expressed in leaves, roots, flowers, petals, shoots, sepals, stolons, and petioles. *StCesA7* and *StCesA8* were highly expressed in shoots, roots, petioles, and stolons but low in flowers, sepals, leaves, and petals. The expression levels of *StCesAs* in different tissues indicate that they have distinct roles in potato growth and development. The differences in gene expression patterns between RNA-seq and qRT-PCR data may be due to various factors, such as different strains, growth conditions, and other external factors (**Figure 11**). The results of qRT-PCR (**Figure 9**) showed that the expressions of *StCesA1*, *StCesA4*, and *StCesA8* were down-regulated with time, and the expressions of *StCesA2* and *StCesA3* reached a high level at 24h, and the expression of *StCesA7* reached a high level at 48h. The expression of *StCesA5* and *StCesA6* reached a high level at 72h. These temporal patterns suggest that the *StCesA* genes play dynamic roles during different phases of potato growth and development.

Comparative studies have shown that the cellulose synthase gene family is highly conserved across different plant species and plays a crucial role in cell wall biosynthesis. For instance, research on barley identified seven *CslF* genes that are involved in the biosynthesis of *β*-d-glucans in cell walls (Burton et al., 2008). Similarly, a genome-wide bioinformatics analysis of the cellulose synthase gene family in common bean revealed distinct expression patterns during pod development (Liu et al., 2022). Moreover, comparative genomics and co-expression networks have been used to analyze the *CesA* gene family in eudicots, providing insights into their evolution and functional diversification. The results showed that duplications within the gene family have contributed to the expansion of *CesA* gene members in eudicots, suggesting an evolutionary mechanism for increasing the complexity and diversity of cellulose synthase functions. Co-expression networks have further demonstrated that primary and secondary cell wall modules are duplicated in eudicots, which may reflect the adaptation of these plants to various environmental conditions and developmental stages (Nawaz et al., 2019).

These results emphasize the complexity nature of cellulose biosynthesis regulation and its influence on plant growth and development. They also highlight the significance of *CesA* genes in diverse physiological functions and their potential as targets for genetic modification to enhance crop characteristics.

## 5 Conclusions

In this study, a total of 8 *StCesA* genes were identified in the whole potato genome using bioinformatics methods. *StCesA* gene family members are scattered on seven different chromosomes and divided into four clusters based on the phylogenetic tree. Intraspecies analysis showed a gene duplication event between *StCesA1* and *StCesA4*. Synteny analysis showed that *StCesAs* and *SlCesAs* shared 10 pairs of homologous genes. We analyzed the expression pattern of *StCesAs* in different tissues in DM potato using RNA-seq data. The RNA-seq analysis showed that *StCesA2* had the highest expression across all tissues, *StCesA6* was weakly expressed in stolons and petals, and *StCesA8* was not expressed in petals. Specifically, *StCesA1* and *StCesA2* had the highest expression in petioles, *StCesA3*, *StCesA7*, and *StCesA8* in stolons, *StCesA4* and *StCesA6* in petals, and *StCesA5* in flowers. Additionally, qRT-PCR analysis showed that the relative expression levels of *StCesA1*, *StCesA4*, and *StCesA7* were down-regulated, and *StCesA5* was up-regulated 72 hours after being infected by *P. infestans*. The results of this experiment are expected to promote the study of the *CesA* gene family and lay the foundation for further research on the role of *CesA* genes in biotic stress.

## Data Availability

Publicly available datasets were analyzed in this study. This data can be found here: PGSC repository: http://spuddb.uga.edu/.
